# Increased expression of CSF1 in patients with eosinophilic asthma

**DOI:** 10.1002/iid3.847

**Published:** 2023-05-10

**Authors:** Lijuan Du, Lu Tang, Lisha Xiao, Kun Tang, Zhimin Zeng, Yuxia Liang, Yubiao Guo

**Affiliations:** ^1^ Department of Pulmonary and Critical Care Medicine The First Affiliated Hospital of Sun Yat‐Sen University Guangzhou Guangdong China; ^2^ Institute of Respiratory Diseases of Sun Yat‐Sen University Guangzhou Guangdong China; ^3^ Department of Respiratory and Critical Care Medicine The Affiliated Hospital of Guizhou Medical University Guiyang Guizhou China

**Keywords:** airway eosinophil inflammation, asthma, CSF1‐CSF1R, STAT1

## Abstract

**Background:**

The link between colony‐stimulating factor 1 (CSF1) and asthma was reported recently. However, the role and mechanism of CSF1 in asthma remain poorly understood. In this study, we aimed to explore the expression and its potential mechanism of CSF1 in asthma.

**Methods:**

CSF1 expression in the airway samples from asthmatics and healthy controls were examined, then the correlations between CSF1 and eosinophilic indicators were analyzed. Subsequently, bronchial epithelial cells (BEAS‐2B) with CSF1 overexpression and knockdown were constructed to investigate the potential molecular mechanism of CSF1. Finally, the effect of CSF1R inhibitor on STAT1 was investigated.

**Results:**

The expression of CSF1 was significantly increased in patients with asthma compared to healthy controls, especially in patients with severe and eosinophilic asthma. Upregulated CSF1 positively correlated with airway‐increased eosinophil inflammation. In vitro, cytokines interleukin 13 (IL‐13) and IL‐33 can stimulate the upregulation of CSF1 expression. CSF1 overexpression enhanced p‐CSF1R/CSF1R and p‐STAT1/STAT1 expression, while knockdown CSF1 using anti‐CSF1 siRNAs decreased p‐CSF1R/CSF1R and p‐STAT1/STAT1 expression. Furthermore, the inhibitor of CSF1R significantly decreased p‐STAT1/STAT1 expression.

**Conclusions:**

Sputum CSF1 may be involved in asthmatic airway eosinophil inflammation by interacting with CSF1R and further activating the STAT1 signaling. Interfering this potential pathway could serve as an anti‐inflammatory therapy for asthma.

## Graphical abstract

Graphical abstract: Potential regulatory mechanism of CSF1 in asthma.

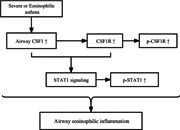

## 1 INTRODUCTION

Asthma is a chronic airway inflammatory disease characterized by airway hyperresponsiveness (AHR) and reversible airflow restriction. Airway epithelial allergic/eosinophilic inflammation mediated by type 2 cytokines, such as interleukin‐4 (IL‐4), IL‐5, and IL‐13, is one of the most common pathological features in asthma.^1^, ^2^, ^3^, ^4^ A range of cells has been associated with asthma progression, including eosinophils, mast cells, macrophages, neutrophils, T lymphocytes, basophils, macrophages/monocytes, dendritic cells, innate lymphoid cells, and epithelium cells.^5^, ^6^ Which, the importance of eosinophils in chronic allergic asthma has been elegantly highlighted.^7^ Numerous evidence indicated that induced sputum‐derived biomarkers, as a noninvasive assessment method, could be used to identify the phenotypes and monitor the progress of asthma.^8^, ^9^, ^10^

Colony‐stimulating factor 1 (CSF1), also known as macrophage‐CSF (M‐CSF), is a kind of cytokine that controls the proliferation, differentiation, and phagocytosis of macrophages.^11^, ^12^ CSF1 and its receptor (CSF1R) play multiple essential roles in the pathogenesis of immune‐mediated inflammatory diseases,^13^, ^14^ such as multiple sclerosis (MS), pigmented villonodular synovitis, rheumatoid arthritis (RA), and interstitial lung disease (ILD).^15^, ^16^, ^17^, ^18^, ^19^ It has been reported that airway epithelial cell‐derived CSF1 and CSF1R be involved in the pathogenesis of asthma, especially in the activation of dendritic cells (DCs) and subsequent allergic inflammation.^20^, ^21^, ^22^, ^23^ Increased CSF1‐induced cytokine production through a direct action of airway type 2 innate lymphoid cells (ILC2s) and enhanced allergic sensitization in house dust mite (HDM)‐exposed animals.^24^

It was reported that CSF1 activates STAT1 transcription factor in bone marrow macrophages.^25^ Binding CSF1 to the wild‐type CSF1R results in the phosphorylation of STAT1 in macrophage cells. The STAT1 signaling pathway is thought to regulate several immune‐mediated diseases, including asthma,^26^, ^27^ by inducing proinflammatory subgroups.^28^ Consistently, in our previous pilot study, based on a sputum microarray expression profile, we found that CSF1 was a key gene in the pathogenesis of asthma,^29^ subsequent research showed CSF1 was significantly increased in severe and eosinophilic asthma, but there was few research focusing on the clinical significance and potential underlying mechanism of CSF1 in BEAS‐2B cells. Therefore, the original point of this study was to further investigate the expression of airway CSF1 in different asthma phenotypes and understand the potential regulatory mechanism of CSF1 in asthma, namely, to discover the regulatory relationship between CSF1, CSF1R, and the STAT1 signaling in bronchial epithelial cells.

In this investigation, we determined the expression of CSF1 and its mechanism role in allergen asthma. We demonstrated that CSF1 expression was significantly increased in asthmatic airway specimens, especially in severe and eosinophilic phenotypes. Increased sputum CSF1 significantly correlated with increased eosinophilic indicators, such as the fraction of exhaled nitric oxide (FeNO), peripheral blood eosinophils percentage (EOS%), and serum total immunoglobulin E (IgE). Our findings demonstrated overexpression of CSF1 significantly increased p‐CSF1R/CSF1R and p‐STAT1/STAT1 expression, while knockdown CSF1 reversed this trend. The data above suggest that sputum CSF1 may be involved in asthmatic airway inflammation by activating its receptor and the STAT1 signaling.

## 2 METHODS

### 2.1 Bioinformatic analysis of CSF1 based on GEO datasets

In our previous pilot study,^29^ we initially found that CSF1 was a key gene in asthma using the data set GSE76262 (21 healthy controls and 118 asthma).^30^ Subsequently, multiomics analysis was used to verify the expression of CSF1 in asthma, including GSE137268 (15 healthy controls and 54 asthma),^31^ GSE45111 (total 47 asthma, including 17 eosinophilic, 12 neutrophilic, and 18 paucigranulocytic),^32^ and GSE74986 (12 healthy controls and 74 asthma).^33^ Based on these microarray datasets, the expression of CSF1 in different subgroups of asthma was further analyzed.

### 2.2 Participants

Participants diagnosed with asthma (without inhaled glucocorticoid therapy, ICS) were recruited from the First Affiliated Hospital of Sun Yat‐Sen University (Guangzhou, China). Asthma was diagnosed according to the Global Initiative for Asthma (GINA) guidelines^34^ as follows: symptoms of wheezing, cough, and dyspnea; histamine (<8 mg/mL) caused forced expiratory volume to decrease 20% fall (PC_20_) in the first second (FEV_1_) and/or increase ≥12% in FEV_1_ after inhalation of 200 μg salbutamol. Healthy controls without respiratory or systemic inflammatory diseases were recruited through advertising. All participants provided written informed consent. The study was approved by the Ethics Committee of the First Affiliated Hospital of Sun Yat‐Sen University (No. 2021(071)).

### 2.3 Collecting induced sputum

Participants were asked to induce sputum production by inhaling a 4.5% sodium chloride aerosol solution under the supervision of a respiratory clinician. Sputum samples should be processed within 2 h after collection. The sputum bolt was then dissolved with 0.1% DL‐Dithiothreitol (DTT; Sigma), a volume four times its weight.^35^ The dissolved solution was filtered through a cell screen and centrifuged (1000*g* 5 min, Eppendorf), the sputum cells in the sediment were collected in 1 mL TRIzol (Invitrogen) for RNA extraction and the sputum supernatant were stored in −80°C refrigerator for enzyme‐linked immunosorbent assay (ELISA) detection. Besides, other clinical information including FeNO, blood EOS%, and serum IgE were collected.

### 2.4 Cell culture and treatment

BEAS‐2B cells were purchased from ATCC (USA) and cultured in Dulbecco's Minimal Essential Medium (DMEM) containing 10% fetal bovine serum. BEAS‐2B cells were stimulated with or without cytokines IL‐13 or IL‐33 (0, 10, 20, 40 ng/mL, respectively; Peprotech),^36^, ^37^, ^38^ transfected with control vector or overexpression CSF1 vector (800 ng/mL; GeneCodex), transfected with small interfering RNA (siRNA) negative control or anti‐CSF1 siRNAs (20 μM; GeneCodex) using transfection reagent Lipofectamine 3000 (Invitrogen), and treated with or without GW2580 (10 μM; Glpbio). Twenty‐four hours later, cells were harvested for subsequent detection. The primers of CSF1 siRNAs were listed in Table 1.

**Table 1:** The primers of anti‐CSF1 siRNAs.

si‐1	GAUCCAGUGUGCUACCUUATT
si‐2	GCUGCUUCACCAAGGAUUATT
si‐3	CAAAGAAUCUCCUUGACAATT

Abbreviations: CSF1, colony‐stimulating factor 1; siRNA, small interfering RNA.

### 2.5 Quantitative real‐time polymerase chain reaction (qRT‐PCR)

Total RNA from induced sputum cells and treated BEAS‐2B cells was isolated using TRIzol reagent (Invitrogen) according to the manufacturer's instructions. Reverse‐transcribed using the Evo M‐MLV RT Premix kit (AG). The expressions of candidate genes were quantitatively determined using Biosystems Light Cycler 480 (Applied Biosystems) as standard procedures. The primers used were as follows. CSF1: forward, 5′‐AGACCTCGTGCCAAATTACATT‐3′, reverse, 5′‐GGTGTCTCATAGAAAGTTCGGA‐3′. CSF1R: forward, 5′‐GGGAATCCCAGTGATAGAGCC‐3′, reverse, 5′‐TTGGAAGGTAGCGTTGTTGGT‐3′. STAT1: forward, 5ʹ‐CAGCTTGACTCAAAATTCCTGGA‐3ʹ, reverse, 5ʹ‐TGAAGATTACGCTTGCTTTTCCT3′. Glyceraldehyde 3‐phosphate dehydrogenase (GAPDH): forward, 5ʹ‐ACCCAGAAGACTGTGGATGG‐3ʹ, reverse, 5ʹ‐TTCTAGACGGCAGGTCAGGT‐3ʹ.

### 2.6 ELISA

CSF1 protein levels in the supernatant of induced sputum were detected using a commercially available ELISA kit (MM‐2022H1, detection limit: 20–640 pg/mL) according to the manufacturer's instructions (MEIMIAN, China, http://www.mmbio.cn/).

### 2.7 Western blot analysis

CSF1, CSF1R, and STAT1 protein levels in the induced sputum cells and BEAS‐2B cells were determined by western blot analysis according to the manufacturer's instructions. Abs to anti‐M‐CSF (1:1000; Abcam, ab233387), MCSF receptor (1:500; Affinity, AF0080), p‐CSF1R (1:500; Affinity, AF4395), STAT1 (1:500; Affinity, AF6300), p‐STAT1 (1:500; Affinity, AF3300), GAPDH (1:3000; Affinity, AF7021), Tubulin β (1:1000; Affinity, AF7011) and goat antirabbit IgG (H + L) HRP (1:4000; Affinity, S0001) were used in this study.

### 2.8 Statistical analysis

GraphPad Prism V.8.3.0 software was used for the statistical analysis. We calculated means ± standard deviation (SD) and used parametric tests (Tukey‐adjusted one‐way analysis of variance or unpaired *t*‐tests) to compare between groups for normally distributed data. And we calculated the median (quartile range) and used nonparametric tests (Kruskal–Wallis test vs. Dunn intergroup comparison or Mann–Whitney test) for nonnormally distributed data. Spearman's rank‐order correlation was used to analyze the correlation. *p* < .05 was considered statistically significant.

## 3 RESULTS

### 3.1 Airway CSF1 was upregulated in asthmatic patients

**Figure 1:**
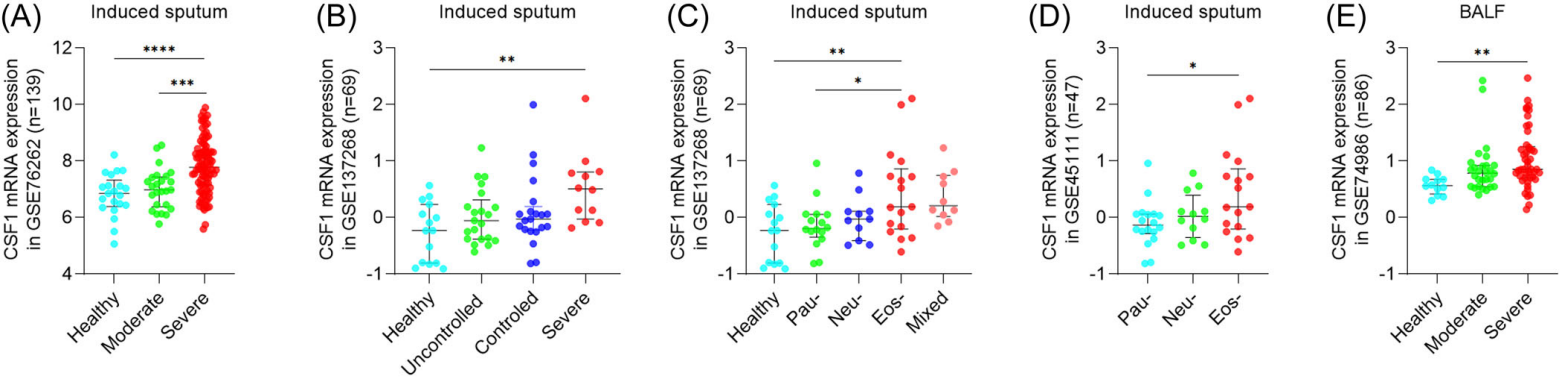
The expression of CSF1 in asthma. (A–E) The mRNA levels of CSF1 were significantly increased in severe asthma and eosinophilic asthma based on microarray datasets (GSE76262, GSE137268, GSE45111, and GSE74986). (One‐way ANOVA followed by Tukey's multiple comparison were used). *p < .05, **p < .01, ***p < .001, ****p < .0001. ANOVA, analysis of variance; CSF1, colony‐stimulating factor 1; Eos‐A, eosinophilic asthma; mRNA, messenger RNA; Neu‐A, neutrophilic asthma; Pau‐A, paucigranulocytic asthma.

In our previous pilot study, we found that sputum CSF1 played a key role in asthma based on GSE76262, subsequent multiomics analysis showed that CSF1 messenger RNA (mRNA) was significantly upregulated in severe and eosinophilic asthma (Figure 1A–E). To further confirm CSF1 expression in patients with asthma from a clinical perspective, we prospectively recruited 25 healthy controls and 72 asthmatic patients for PCR and ELISA detection (Table 2).

**Table 2:** Characteristics of participants.

	Healthy controls	Asthmatics	*p* Value
	(*n* = 25)	(*n* = 72)	
Age (years)	36.69 ± 15.89	42.43 ± 15.57	.036
Sex, M:F (%F)	11:14 (31.8%)	42:30 (68.2%)	.249
BMI (kg/m^2^)	21.9 ± 3.36	23.2 ± 2.96	.038
Histamine PC20, mg/mL	‐	2.19 ± 2.37	‐
FEV_1_, % predicted	98.26 ± 19.44	82.18 ± 22.58	<.0001
FVC, % predicted	99.09 ± 17.67	92.62 ± 20.18	.158
FEV1/FVC, %	84.83 ± 10.13	72.66 ± 13.43	<.0001
FeNO (ppb)	16.59 ± 6.95	56.1 ± 47.44	<.0001
Blood eosinophils (10^9^/L)	0.19 ± 0.30	0.31 ± 0.29	.073
Blood eosinophils (%)	2.79 ± 3.62	4.74 ± 4.20	.032
IgE (IU/mL)	101.3 ± 98.67	212.7 ± 08.3	.007

*Note*: Values were presented as mean ± standard deviation.

Abbreviations: BMI, body mass index; FEV1, forced expiratory volume in the first second; FeNO, fraction of exhaled nitric oxide; FVC, forced vital capacity; IgE, serum total immunoglobulin E.

**Figure 2:**
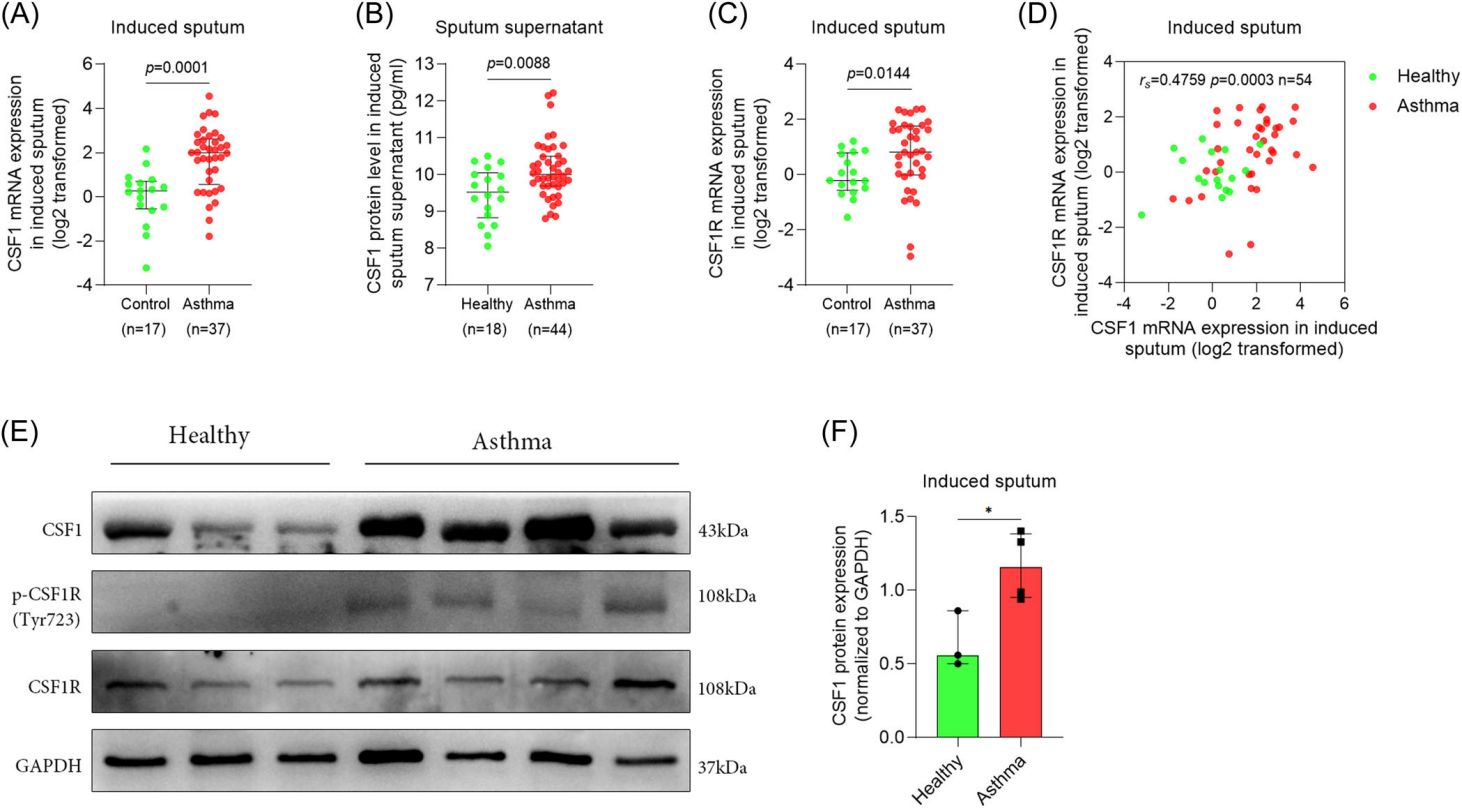
The expression of CSF1 and its receptor in asthma. (A) The mRNA levels of CSF1 in the induced sputum of healthy controls (*n* = 17) and asthma patients (*n* = 37) were detected by qRT‐PCR; (B) The protein levels of CSF1 in the supernatant of induced sputum of healthy controls (*n* = 18) and asthma patients (*n* = 44) were measured by ELISA; (C) The mRNA levels of CSF1R in the induced sputum of healthy controls (*n* = 17) and asthma patients (*n* = 37) were detected by qRT‐PCR; (D) The correlation between the mRNA levels of CSF1 and CSF1R in the induced sputum of subjects; (E) The protein levels of CSF1 and its receptor in the induced sputum (three healthy controls and four asthmatics) were analyzed by western blot analysis; (F) Densitometry assay of CSF1 sputum western blot analysis results was analyzed using ImageJ. (Mann–Whitney test and Spearman's rank‐order correlation were used). *p < .05. CSF1, colony‐stimulating factor 1; ELISA, enzyme‐linked immunosorbent assay; GAPDH, glyceraldehyde 3‐phosphate dehydrogenase; mRNA, messenger RNA; qRT‐PCR, quantitative real‐time polymerase chain reaction.

First, we recruited 17 healthy controls and 37 asthmatic patients to detect the expression of CSF1 mRNA in the induced sputum cells by qRT‐PCR. Results showed that CSF1 mRNA expression was significantly increased in asthmatic patients compared to healthy controls (*p* = .0001) (Figure 2A), which was consistent with microarray analysis. Furthermore, we enrolled 18 healthy controls and 44 asthma patients to detect the expression of CSF1 protein in the supernatant of induced sputum by ELISA. Also, the results showed that CSF1 protein levels were significantly increased in asthmatic patients (*p* = .0088) (Figure 2B), which was consistent with the mRNA expression results. Besides, we also detect the mRNA expression of its receptor (CSF1R), our results showed CSF1R was increased in asthma (*p* = .0144) (Figure 2C), A positive correlation between CSF1 and CSF1R was observed (*r*_s_ = 0.4759, *p* = .0003) (Figure 2D). Meanwhile, a western blot was conducted to analyze the expression of the CSF1 protein and its receptor in the induced sputum cells. Our results showed that both CSF1 and its receptor were significantly increased in asthma compared to healthy participants (Figure 2E,F). The data above showed elevated airway CSF1 may play a critical role in the pathogenies of asthma.

### 3.2 CSF1 was positively associated with eosinophil inflammation

**Figure 3:**
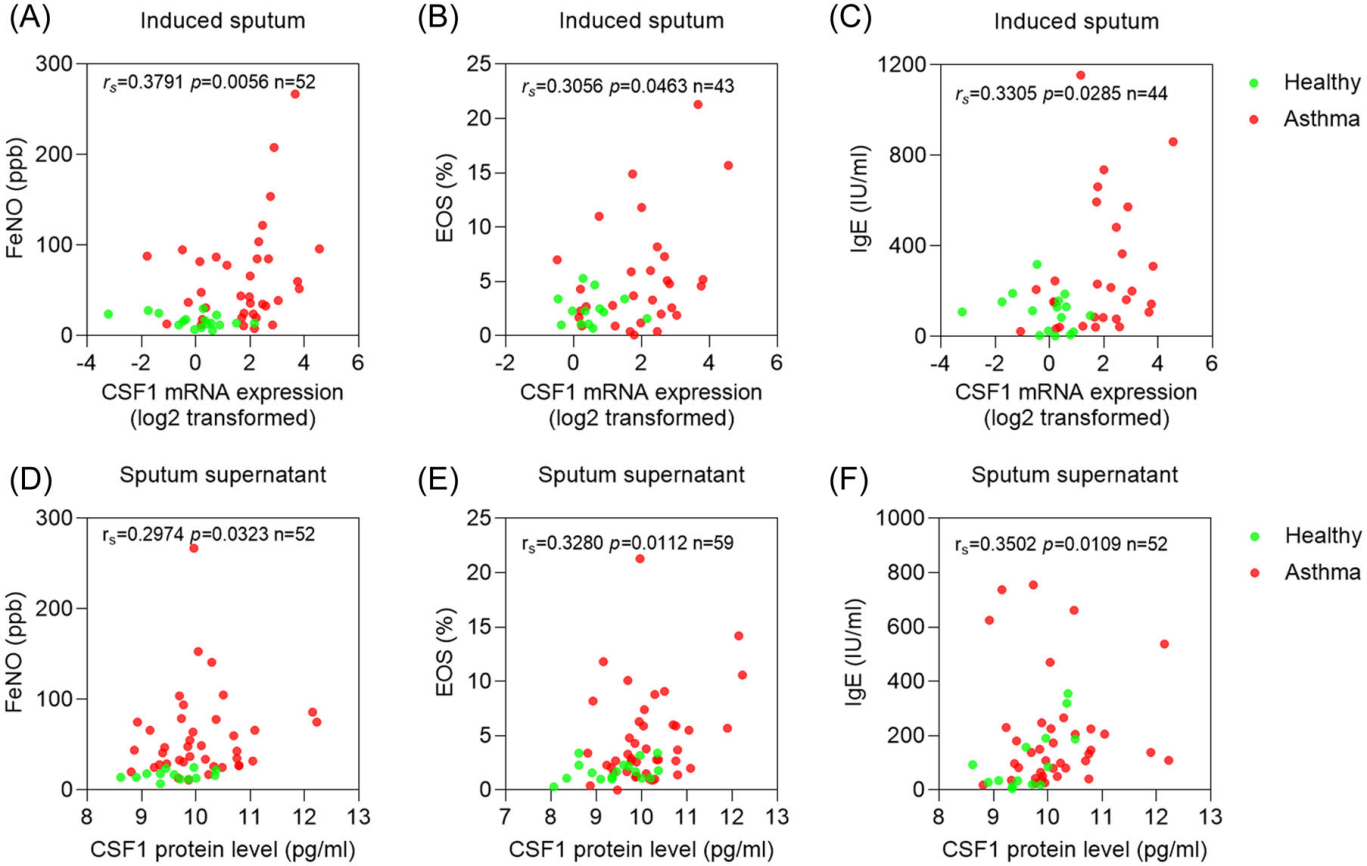
CSF1 was associated with airway eosinophilic inflammation. (A–C) The correlation between the mRNA levels of CSF1 and FeNO, EOS% and IgE; (D–F) The correlation between the protein levels of CSF1 and FeNO, EOS%, and IgE. (Spearman's rank‐order correlation was used). CSF1, colony‐stimulating factor 1; EOS%, peripheral blood eosinophils percentage; FeNO, fraction of exhaled nitric oxide; IgE, serum total immunoglobulin E; mRNA, messenger RNA.

It has been well known that FeNO, blood EOS%, and IgE are significant predictors for airway eosinophil and type 2 inflammation in allergic asthma.^39^, ^40^, ^41^, ^42^, ^43^, ^44^ As we found CSF1 was significantly increased in asthma with an eosinophilic phenotype, we further explored the correlation between CSF1 and airway eosinophilic inflammation indicators. Our results showed that upregulated CSF1 mRNA levels significantly correlated with increased FeNO (*r*_s_ = 0.3791, *p* = .0056), blood EOS% (*r*_s_ = 0.3056, *p* = .0463), and IgE (*r*_s_ = 0.3305, *p* = .00285) (Figure 3A–C). Consistently, the positive correlations between CSF1 protein levels (detected by ELISA) and FeNO (*r*_s_ = 0.2974, *p* = .0323), EOS% (*r*_s_ = 0.3280, *p* = .0112), and IgE (*r*_s_ = 0.3502, *p* = .0109) were also observed (Figure 3D–F). In conclusion, elevated airway CSF1 may be involved in the pathogenesis of airway‐increased eosinophil inflammation in asthma.

### 3.3 Overexpression of CSF1 enhanced CSF1R and STAT1 expression in BEAS‐2B cells

**Figure 4:**
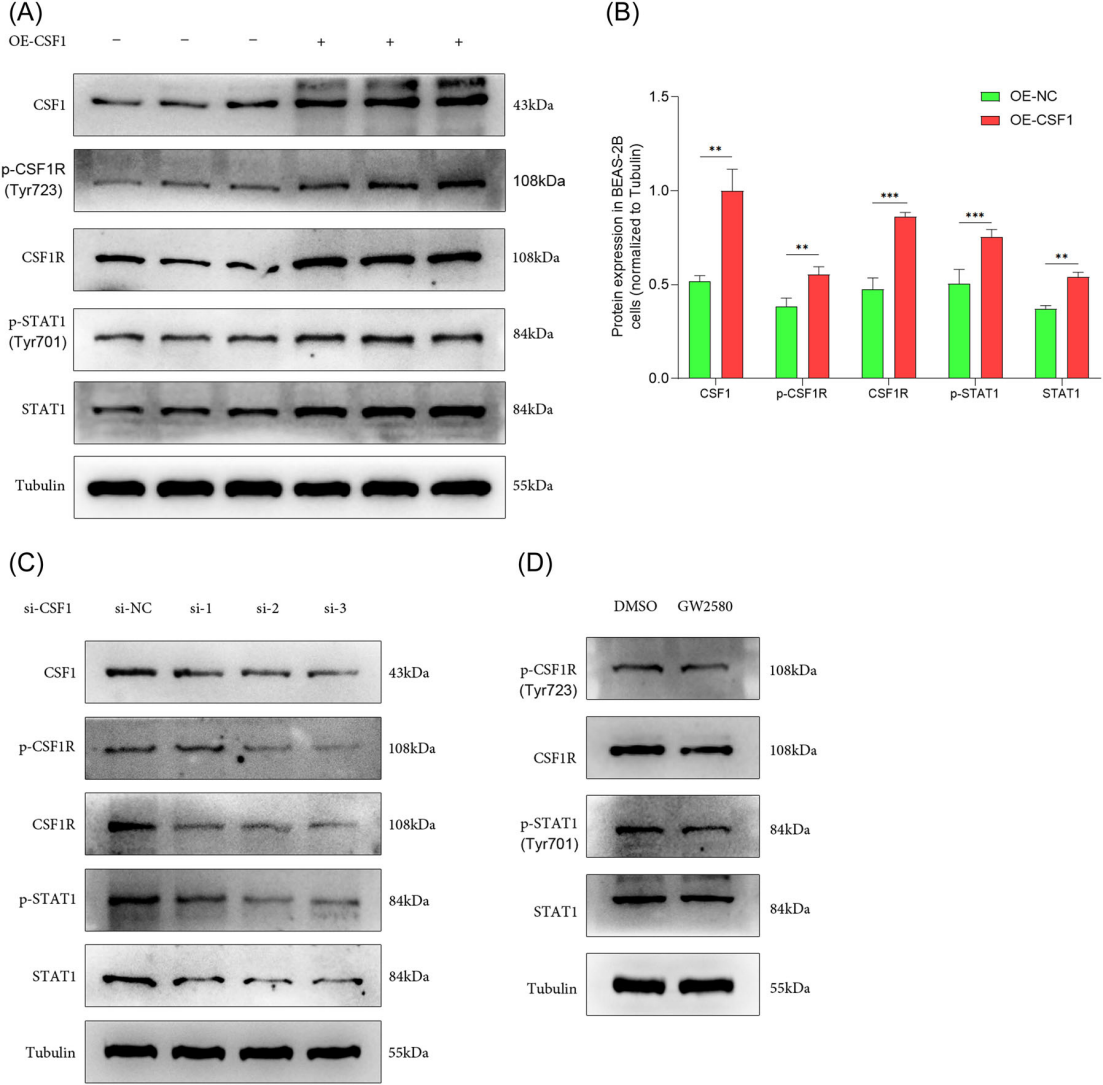
Overexpression of CSF1 activated its receptor and STAT1 protein. (A) CSF1 overexpression vector and control empty vector were transfected BEAS‐2B cells, then the protein levels of CSF1, p‐CSF1R/CSF1R, and p‐STAT1/STAT1 were measured by western blot analysis; (B) Densitometry assay of CSF1 sputum western blot analysis results was analyzed using ImageJ; (C) CSF1 siRNAs and siRNA negative control were transfected BEAS‐2B cells, then the protein levels of CSF1, p‐CSF1R/CSF1R, and p‐STAT1/STAT1 were measured by western blot analysis; (D) the effect of the inhibitor of CFS1R on STAT1 expression was detected by western blot analysis (unpaired *t*‐test was used). **p < .01, ***p < .001. CSF1, colony‐stimulating factor 1; siRNA, small interfering RNA; STAT1, signal transducer and activator of transcription 1.

CSF1R, the receptor for CSF1, was reportedly involved in the pathogenesis of asthma.^21^, ^22^ By detecting CSF1R mRNA in the induced sputum cells, we found that CSF1 was positively correlated with CSF1R expression, suggesting that increased CSF1 may be involved in asthmatic airway eosinophil inflammation through interacting with CSF1R. To further investigate whether CSF1 affects the expression of CSF1R, we established an overexpression vector of CSF1 to transfect BEAS‐2B cells for 24 h. Our results showed that overexpression of CSF1 significantly enhanced the protein expression of CSF1R and the phosphorylation of CSF1R (Figure 4A,B), while knockdown CSF1 significantly decreased the expression of p‐CSF1R/CSF1R compared to siRNAs negative control (Figure 4C).

Then, we also demonstrated that STAT1 protein was activated in response to CSF1 in BEAS‐2B cells, specifically, the protein expression of STAT1 and the phosphorylation of STAT1 was increased in response to overexpression of CSF1 (Figure 4A,B), but decreased after knockdown of CSF1 in BEAS‐2B cells (Figure 4C). Moreover, the selective CSF1R inhibitor GW2580 also inhibited the expression of STAT1 in BEAS‐2B cells (Figure 4D), which further indicated that the STAT1 signaling is one of the downstream molecules of CSF1‐CSF1R.

**Figure 5:**
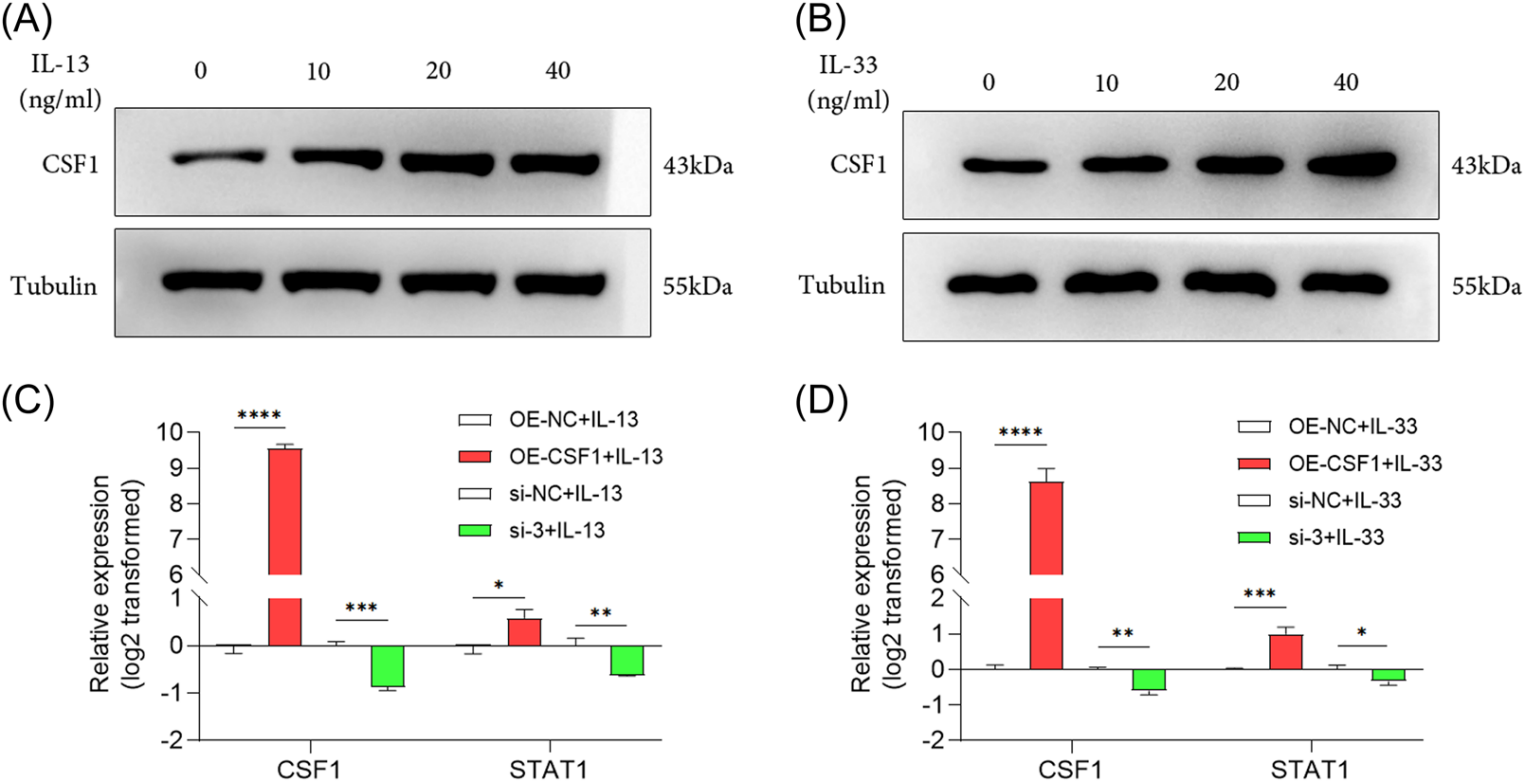
The effects of cytokines IL‐13 and IL‐33 on CSF1 and STAT1 expression. (A, B) The protein levels of CSF1 in BEAS‐2B cells treated with cytokines IL‐13 and IL‐33 at different concentrations (0, 10, 20, and 40 ng/mL) for 24 h; (C, D) The effects of CSF1 on STAT1 in BEAS‐2B cells stimulated with IL‐13 or IL‐33 cytokines were detected by qRT‐PCR. (One‐way ANOVA followed by Tukey's multiple comparison test was used). *p < .05, **p < .01, ***p < .001, ****p < .0001. ANOVA, analysis of variance; BEAS‐2B, bronchial epithelial cells; CSF1, colony‐stimulating factor 1; IL‐13, interleukin 13; STAT1, signal transducer and activator of transcription 1; qRT‐PCR, quantitative real‐time polymerase chain reaction.

Furthermore, we detected CSF1 protein in BEAS‐2B cells under the stimulation of cytokines IL‐13 and IL‐33 at different concentrations for 24 h. Our results showed that CSF1 could be induced by these cytokines (Figure 5A,B). Subsequently, we verified the expression of STAT1 in response to CSF1 from a transcriptome perspective. Specifically, in BEAS‐2B cells stimulated with either IL‐13 (20 ng/mL) or IL‐33 (40 ng/mL), CSF1 positively induced STAT1 expression via regulating CSF1 expression (Figure 5C,D). These results suggest that airway CSF1 may interact with CSF1R to further activate the STAT1 signaling and participate in airway eosinophil inflammation in asthma.

## 4 DISCUSSIONS

CSF1, as one of the inflammation‐related genes, plays a critical role in a variety of inflammatory diseases,^45^, ^46^, ^47^ but there were few reports describing CSF1 expression in the airway and its clinical implications in asthma. We previously studied CSF1, a key gene in asthma in the induced sputum from the GSE76262 data set.^29^ In this study, we initially demonstrated CSF1 was significantly increased in patients with severe and eosinophilic asthma, subsequent validation showed that both mRNA and protein expression of CSF1 were significantly upregulated in the induced sputum of asthmatics. Increased CSF1 was correlated with increased FeNO, blood EOS%, and serum IgE. Besides, increased CSF1 was observed in BEAS‐2B cells stimulated with cytokines IL‐13 or IL‐33. Furthermore, a positive correlation between CSF1 and its receptor in the induced sputum was observed, overexpression of CSF1 significantly elevated the expression of CSF1R, as well as STAT1, while knockdown of CSF1 decreased CSF1R and STAT1 expression. The data above indicate that upregulated airway CSF1 may be involved in the airway inflammation of asthma by interacting with its receptor and activating the STAT1 protein.

Epithelial cell‐derived CSF1 has been reported to be involved in airway allergic disease^48^, ^49^ and participated in the IgE production response to allergen asthma.^20^ CSF1 along with Th2 cytokines IL‐3 and IL‐5 could regulate the differentiation and function of eosinophils and further participate in the resolution of inflammation.^50^, ^51^ Recent studies showed that eosinophils contribute to the development of severe asthma or asthma acute exacerbations.^52^, ^53^ The accelerated decline of FEV1 in asthmatic patients presented the increase of eosinophils, IL5, and IL8 in the induced sputum.^54^ Here, we found that CSF1 expression was increased in asthmatics with a severe and eosinophilic phenotype, and CSF1 mRNA and protein expression were positively correlated with clinical eosinophilic indicators (FeNO, EOS%, and IgE). In addition, IL‐13 and IL‐33 induced the elevation of CSF1 in BEAS‐2B cells. These results suggest that airway CSF1 may be involved in Th2 cytokines‐mediated asthmatic airway eosinophil inflammation.

CSF1R, namely the CSF1 receptor, plays an important role in innate immunity by regulating the survival, proliferation, differentiation, and chemotaxis of macrophages.^55^, ^56^ CSF1R expression was increased in HDM‐induced experimental asthma,^23^ and its gene polymorphism was associated with increased asthma risk, in which, CSF1R + 22693T>C may be predisposed to asthma.^21^ Our findings showed that CSF1R was significantly upregulated in asthmatic patients, which provided further evidence for previous research. Beyond that, we found a positive correlation between CSF1 and CSF1R in the induced sputum. It was reported that rhCSF1 treatment further increased the expression of total‐CSF1R and p‐CSF1R in neonatal hypoxic‐ischemic rats.^57^ The CSF1/CSF1R pathway plays a key role in delivering allergens to regional lymph nodes through the activation of dendritic cells.^22^ To further investigated the underlying regulated effects of CSF1 on CSF1R, we constructed the overexpression/knockdown models of CSF1 in BEAS‐2B cells. Similarly, we observed CSF1 overexpression significantly promoted CSF1R protein and the phosphorylation of CSF1R compared to control, while CSF1 knockdown inhibited p‐CSF1R/CSF1R expression, which further elucidated the positive correlation between CSF1 and CSF1R. The findings indicated that CSF1 may exert its modulatory role by activating CSF1R in the airway pathogenesis of asthma.

Dendritic cells are a special type of antigen‐presenting cell that link innate and adaptive immune system function. And the activation of dendritic cells is considered one of the key factors in causing allergic airway inflammation.^58^, ^59^, ^60^ Jackson et al. showed that activation of the STAT pathways plays a role in the differentiation and maturation of dendritic cells (DCs), and the optimal activation of STAT1 in DCs maturation requires the combined action of IL‐4 and CSF1.^61^ To further explore the effects of CSF1 on the STAT1 signaling in BEAS‐2B cells stimulated with IL‐13/IL‐33 or not, we detected the p‐STAT1/STAT1 expression through overexpression/knockdown of CSF1. Positive feedback from CSF1 to STAT1 was observed, specifically, we proved that CSF1 overexpression significantly promoted STAT1 expression but anti‐CSF1 siRNAs significantly downregulated STAT1 expression whether IL‐13/IL‐33 was treated or not. Furthermore, the inhibitor of CSF1R inhibited the STAT1 protein and the phosphorylation of STAT1. Thus, we presumed that CSF1‐CSF1R is associated with airway eosinophil inflammation might via activating the STAT1 signaling in asthma.

According to the above results, it could be speculated that increased CSF1 in asthma may interact with CSF1R to further activate the STAT1 signaling pathway, which plays a key role in the pathogenesis of airway inflammation in asthma. Hence, targeting CSF1‐CSF1R‐STAT1 may bring novel insights into allergic asthma therapy. It has been reported that CSF1 antagonist treatment could ablate local inflammation.^62^ Targeting CSF1R using a CSF1R inhibitor exerted therapeutic effects on HDM‐induced allergic airway inflammation.^23^ In vivo, CSF1/CSF1R intervention prevents allergen sensitization and subsequent Th2 allergic inflammation.^22^ The therapeutic effect of CSF1‐CSF1R‐STAT1 signaling pathway intervention on asthma needs to be further studied.

Our study had some limitations. First, our sample of asthma was insufficient for subgroup analysis. Second, the patients in this study were newly diagnosed with asthma (without ICS treatment), and the lack of follow‐up data led to the loss of CSF1 expression in patients with asthma after ICS treatment. Third, due to the difficulty to obtain and culture eosinophils, we failed to explore the role and mechanism of CSF1 in eosinophils. Finally, we have not yet demonstrated in‐depth mechanism and functional verification in vivo in a model of eosinophilic asthma.

## 5 CONCLUSIONS

In this study, we found that airway CSF1 was significantly elevated in patients with asthma, especially in asthma with a severe and eosinophilic phenotype, and increased CSF1 was associated with increased asthmatic airway eosinophil inflammation. In addition, CSF1 can interact with CSF1R to regulate the expression of STAT1 in BEAS‐2B cells. These findings indicated that increased airway CSF1 might play a key role in asthmatic airway eosinophil inflammation via interacting with its receptor further activating the STAT1 signaling pathway. Therefore, interference with this potential pathway has positive implications for asthmatic treatment.

## AUTHOR CONTRIBUTIONS

**Lijuan Du**: Data curation; formal analysis; investigation; methodology; project administration; software; validation; writing—original draft. **Lu Tang**: Data curation; investigation; methodology; validation; writing—original draft. **Lisha Xiao**: Data curation; investigation; validation. **Kun Tang**: Data curation; investigation; software; supervision; validation. **Zhimin Zeng**: Conceptualization; funding acquisition; supervision. **Yuxia Liang**: Conceptualization; project administration; resources; supervision; writing—review and editing. **Yubiao Guo**: Conceptualization; funding acquisition; supervision; writing—review and editing.

## CONFLICT OF INTEREST STATEMENT

The authors declare no conflict of interest.

## ETHICS STATEMENT

This study was approved by the Ethics Committee of the First Affiliated Hospital of Sun Yat‐Sen University (No. 2021(071)). All participants provided written informed consent.

## Data Availability

The data that support the findings of this study are available from the corresponding author upon reasonable request.
